# Feel Safe to Take More Risks? Insecure Attachment Increases Consumer Risk-Taking Behavior

**DOI:** 10.3389/fpsyg.2019.00874

**Published:** 2019-04-24

**Authors:** Yuanyuan Jamie Li, Su Lu, Junmei Lan, Feng Jiang

**Affiliations:** ^1^Department of Marketing and E-Commerce, Nanjing University, Nanjing, China; ^2^Department of Human Resource Management and Organizational Behavior, University of International Business and Economics, Beijing, China; ^3^Department of Organization and Human Resources Management, Central University of Finance and Economics, Beijing, China

**Keywords:** attachment styles, consumer behavior, life history, risk-sensitivity, risk-taking

## Abstract

Attachment styles, originated from early childhood experience, have been documented to influence human behaviors among adults. Drawing on life history theory, we examined whether or not, and how, attachment styles impact risk-taking behaviors beyond evolutionary valid domains, and explored the moderation role of parental status. In the consumer behavior context, three correlational studies provide convergent evidence that insecurely attached (vs. securely attached) consumers are more risk-taking in consumption situations like dining in a toilet-themed restaurant or buying genetically modified products. Specifically, insecurely attached consumers were more likely to take risks in two experiential purchase scenarios (Study 1) and this effect was not domain-specific (Study 2). In Study 3, we showed that safety perception mediated the relationship between attachment insecurity and risk-taking, which was manifested by purchase intentions toward genetically modified products. Specifically, insecurely attached individuals perceived genetically modified products to be safer and were more willing to make a purchase. Additionally, parental status moderates the relationship (Studies 2 and 3). We conclude with a discussion on the implications of attachment theory on consumer risk-taking behaviors from a life history perspective.

## Introduction

Drawing on the central assertion that the ways adults perceive and treat their partners and relationships are shaped by their early experiences with their caregivers, attachment theory has greatly enriched our understanding of consumers’ relational consumptions (Simpson et al., 2012), including how people react to advertising strategies depicting interpersonal relationships (David and Bearden, 2017), how they respond to pricing strategies customized for them (David et al., 2017), and how they are influenced by brand personality and brand relationship (Ahluwalia et al., 2009; Thomson et al., 2012).

Nonetheless, many of these studies focus on proximate reasons for consumer behavior, whereas the more ultimate reasons are left unresolved (Simpson and Gangestad, 2001; Simpson et al., 2012). To address this issue, there has been a recent surge of understanding consumer behavior from an evolutionary perspective (e.g., Buss and Schmitt, 1993; Gangestad and Simpson, 2000; Kenrick et al., 2010). Through the same lens, the current study extends consumption implication of attachment theory beyond the relational domain by arguing that adult attachment styles, together with parental status, could influence how people make risky consumption decisions. To this aim, we draw on the life history model of attachment and risk-sensitivity theory within the evolutionary frameworks.

### Attachment Styles and Life History Strategies

Within the framework of life history theory, the life history model of attachment was developed to account for how early childhood environment links to attachment patterns, and reproductive strategies (Draper and Harpending, 1982; Belsky et al., 1991; Chisholm, 1993; Del Giudice, 2009; Chen and Chang, 2012; Chen, 2017; Szepsenwol and Simpson, 2019). Specifically, children who experience supportive and warm parenting and a favorable family environment establish a secure attachment style, and their reproductive strategy in adulthood is characterized by late maturation, long-term mating orientation, and high parental investment (i.e., slow life history strategies). Alternatively, children who constantly experience unfavorable family conditions with inconsistent or irresponsive parenting in their early environment develop an insecure attachment style, and their reproductive strategy in adulthood is characterized by early maturation, short-term mating orientation, and low parental investment (i.e., fast life history strategies). These reproductive strategies are shaped by individuals’ childhood environmental conditions, through the mediation of attachment, and serve to maximize reproductive success (Chisholm, 1993).

Indeed, insecurely attached adolescents have been shown to undertake sexually risky behaviors and drive recklessly (Bogaert and Sadava, 2002; Taubman-Ben-Ari and Mikulincer, 2007). Attachment insecurity is associated with lower condom use, more sexually transmitted diseases (Bogaert and Sadava, 2002), a higher rate of unprotected sex among the HIV-positive (Ciesla et al., 2004), and a higher rate of unplanned pregnancy (Cooper et al., 1998; Feeney et al., 2000).

### Risk Taking as a Fast Life History Strategy

Although these risky behaviors bear high cost including physical injuries, diseases and shortened life expectancy, they could be adaptive responses in the sense of increasing the chance of mating success (Mishra and Lalumière, 2008). In other words, these risky behaviors bear important evolutionary value. According to life history theory, individuals develop different life history strategies based on harshness, and stochastic variation in salient environmental conditions (Ellis et al., 2009). Individuals being reared in environments that vary unpredictably, such as inconsistent responses from the parents, are not able to forecast future maturation reliably from their current situation (Belsky et al., 2012). In other words, they are unable to gain a clear understanding of cause-effect relationships and find it difficult to think of potential negative consequences for their behaviors (Ross and Hill, 2002). Therefore, it is not wise for them to invest in a long-term sense or develop slow life history strategies (Belsky et al., 2012). Instead, they adopt fast life history strategies by putting less weight on the delayed benefits of risk-aversion than on the immediate benefits of risk-taking. This has been manifested by the robust association of present orientation, impulsivity, and a short subjective life expectancy with increased risk taking at the individual level (e.g., Hill et al., 1997; Kahn et al., 2002; Brezina et al., 2009; Wang et al., 2009; Dunkel and Decker, 2010; Chen and Vazsonyi, 2011).

The coexistence of behavioral types, the consistency of behavior through time, and the structure of behavioral correlations across contexts enable us to develop consistent behavioral patterns that can be considered stable individual differences concerning risk taking (Wolf et al., 2007). Therefore, we suggest that individuals will generalize their life history strategies beyond evolutionarily valid domains of risk (e.g., mating). Hence, we predict that,

***H1****:* Relative to securely attached individuals, insecurely attached individuals are more risk-taking.

### Modulation of Parental Status

One important assumption of life history theory is that individuals make different trade-offs at different stages of life (e.g., Chang, 2018; Chang and Lu, 2018). In the domain of risk-taking, this suggests that they make risky choices at different times in life. Some decisions involve more risk than others because they involve greater variance in potential outcomes (Friedman and Savage, 1948; Weber et al., 2004). Therefore, choosing a flipping coin to get either 20 dollars or nothing, is deemed riskier than choosing a sure gain of 10 dollars. In the current study, we examined the effect of a life-history variable, namely parental status (Wang et al., 2009), on risk taking propensity. Parenting is one of the most prominent needs throughout one’s life span (Kenrick et al., 2010), and investing in parenting (i.e., caring for existing offspring) often means fewer resources can be devoted to mating (i.e., creating new offspring). Therefore, parental status has been used in previous research as an index of resource requirements (Wang et al., 2009).

Will being a parent make a consumer more risk-taking? To answer this question, we draw on the risk-sensitivity theory. The risk-sensitivity theory posits that individuals make risk-sensitive decisions contingent on needs—they are more likely to take risks if they are unlikely to meet their needs through safer, low-risk means (for reviews, see Kacelnik and Bateson, 1996, 1997; Mishra and Fiddick, 2012; Mishra et al., 2017). In situations of need, where there is disparity between individuals’ actual state and desired state of resources, people shift from risk-aversion to risk-seeking, because risky choices offer a chance, although slim, of meeting the needs. This prediction has been experimentally demonstrated by studies showing that young adults shifted from risk-aversion to risk-proneness in situations of high need (Mishra and Lalumière, 2010) and in the face of cues suggesting relative competitive disadvantage (Mishra et al., 2014). Given parenting means an increased desired state of need, such as extra knowledge, skills, and social support to cope with parenting demands, being a parent could induce a state of high need and, hence, result in more risk-taking behaviors. A recent large-scale empirical study using multi-national data from the World Value Survey showed that being a parent did actually make a person more risk-taking (Canale et al., 2018).

Based on the above reasoning, we further argue that a high need situation triggered by one’s parental status should be perceived differently from the perspectives of securely and insecurely attached individuals. This is because compared with securely attached individuals, insecure attached individuals expect themselves to be more easily annoyed by children, be stricter or harsher, be less warm toward children, and be generally less confident of their ability to relate to children (Rholes et al., 1997). This lack of initial commitment to one’s parental role and more negative working models might guide their behavioral and affective responses during child rearing. Their inability to perceive and seek social support might further exacerbate their frustration (Florian et al., 1995; Anders and Tucker, 2000). Indeed, compared with securely attached parents, insecurely attached parents enact or experience more negative parenting behaviors, emotions, cognitions (for a review, see Szepsenwol and Simpson, 2019), and higher levels of parenting stress (Abidin, 1992; Deater-Deckard, 2004; Rholes et al., 2006; Nygren et al., 2012). Therefore, insecurely attached parents could perceive their parenting role as more resource-demanding, which might trigger a more significant shift from risk-aversion to risk-proneness. Hence, we predict that,

***H2***: Relative to securely attached individuals, the effect of parental status on risk taking is more significant for insecurely attached individuals.

### The Mediating Role of Safety Perception

Individuals adopting different life history strategies tend to perceive the same risky situation differently. individuals with slow life history strategies tend to be more sensitive to potential physical danger, contamination by pathogens and social exclusion (Nesse, 2004; Nesse and Jackson, 2006). In contrast, individuals with fast life history strategies tend to lower their sensitivity to threats, dangers, and social feedback, given that these signals of threat can be an asset, rather than a weakness (Korte et al., 2005; Del Giudice et al., 2011; Del Giudice, 2014). Therefore, it is reasonable to infer that individuals adopting fast life strategies should perceive the same risky situation as safer than those adopting slow life strategies. Research concerning individuals’ belief about future unpredictability lend primary support to these propositions. Individuals with insecure attachment tend to believe that the world is chaotic and untrustworthy due to their early experiences with caregivers (Ross and Hill, 2002). Accordingly, they focus on current gains over future losses, so that they discount the future and cannot stand delayed gratification (Hill et al., 2008). Stemming from this reasoning, we suggest that their perception of safety is distorted and exaggerated because when evaluating the safety of a decision, they overemphasize the pros of a choice and neglect the cons, which leads to reckless decisions. In this sense, safety perception of insecurely attached individuals is less conservative than securely attached individuals.

From a decision-making perspective, consumer risk taking is the process of choosing from consumption choices that involve different probabilities of potential loss (Dowling and Staelin, 1994), which makes it highly relevant to new product consumption, such as novel experience, and genetically modified (GM) product (Mitchell, 1999). Consumers will perceive a choice to be risky if they expect that an option involves more costs than benefits (Taylor, 1974). Otherwise, they will perceive the choice to be a safe one. The contention that people take more risks when they feel safe is supported by risk compensation theory (Adams and Hillman, 2001) and empirical findings (Weber and Hsee, 1998; Levav and Argo, 2010; Gamble and Walker, 2016). For example, one study showed that when an experimenter patted the participants on their shoulders, they showed increased feelings of safety, and willingness to undertake financial risk (Levav and Argo, 2010). Additionally, participants who wore a baseball cap, introduced as an eye tracker head mount, showed greater willingness to undertake financial risk, presumably because the cap served as a helmet, conveying a feeling of security (Gamble and Walker, 2016). Therefore, we expect safety perception to play a mediating role, leading to a third hypothesis:

***H3*:** Relative to securely attached individuals, insecurely attached individuals perceive higher safety levels and are hence more risk-taking.

### Overview

In the present study, we sought to examine whether insecurely attached individuals take more risks because they feel it is safer, and whether being a parent could amplify this tendency. We argue that life history strategies pertaining to risk-taking could generalize beyond evolutionarily valid domains of risk, in the current case, consumer risk-taking. We tested the effects of attachment styles (i.e., insecure, secure) on risk taking in three correlational studies. In Study 1, we explored the relationship between attachment style and risk taking as indicated by their intention to purchase two new experiential products. In Study 2, we linked attachment styles to domain-specific risk-taking propensities. In Study 3, we used purchase intention of GM products as another manifestation of risk-taking.

This study extends previous research in several ways. First, we are among the first to examine the effect of adult attachment on consumer risk-taking within the life history framework. Second, we use measures of risk taking – purchase intention of experiential products, genetically modified products – that are ecologically valid in the consumer behavior field. Third, we demonstrate the mediating role of safety perception by showing that insecurely attached individuals perceive a risky option to be safer and hence take more risks than securely attached individuals. Finally, we show that, compared with securely attached individuals, being a parent will magnify risk-proneness of insecurely attached individuals to a greater extent.

## Study 1: Risk-Taking in New Experiential Product Consumption

Experiential purchases are made with the primary objective of enjoying the experience or making a memory (e.g., movies, concerts, festivals, tours and travel; Van Boven and Gilovich, 2003; Carter and Gilovich, 2012). They may have a greater downside risk in terms of purchase dissatisfaction (Diehl et al., 2016). Therefore, we render it an adequate manifestation of consumer risk-taking.

### Methods

#### Participants

We paid a participation fee to 103 adult participants from Amazon Mechanical Turk (*Mturk*; *M*_age_ = 33.27, *SD*_age_ = 11.86; 54 men). Before the study, all participants signed an informed consent form.

#### Measures and Procedure

Participants completed a computerized online questionnaire regarding their reactions to two risk-taking scenarios, their dispositional attachment orientations (Brennan et al., 1998), and their basic age and gender demographics.

##### Purchase intention

Participants first indicated their likelihood of engaging in two risky scenarios: willingness to try a bizarre restaurant and to participate in a new gambling game. Likelihoods were indicated on a seven-point scale, anchored by *very unlikely* (1) and *very likely* (7). We counterbalanced the sequence of the two scenarios. The average score of the two items formed the dependent variable (Cronbach’s α = 0.64). Higher scores indicated higher tendencies to take risks.

##### Scenario 1

Modern Toilet Restaurant is a new toilet-themed restaurant where diners sit on acrylic toilets and meals are served in miniature toilet bowls. Previous customers and some famous gourmands have given the restaurant negative feedback.

##### Scenario 2

XEN Betting Ltd., an international betting company, has launched a completely new online video gambling game offering unstable winning probabilities. You can try this new game.

##### Attachment style

Attachment styles were measured by the Experiences in Close Relationship Scale (ECR; Brennan et al., 1998). Participants self-reported how extensively they agreed with 36 items about themselves, ranging from 1 (*disagree strongly*) to 7 (*agree strongly*). The scale includes anxiety (e.g., “I worry about being abandoned.”) and avoidance (e.g., “I avoid showing a partner my deepest feelings”) dimensions. Each dimension has 18 items. We computed mean scores of the two dimensions for each participant (*Cronbach’s*α_anxiety_ = 0.95; *Cronbach’s*α_avoidance_ = 0.91; *r* = 0.59, *p* < 0.05). Higher scores indicated high anxiousness and avoidance. The scores allowed us to identify two types of participants:^1^ securely attached individuals who scored no higher than 4 on both dimensions, and insecurely attached individuals who scored higher than 4 on either dimension. Thirty-one participants were classified as securely attached; 72 were classified as insecurely attached.

### Results and Discussion

A univariate analysis testing the effect of attachment styles (0, insecure attachment; 1, secure attachment) on risk taking tendencies, controlling for age and gender, revealed that insecurely attached participants (*M* = 4.22, *SD* = 1.79) were significantly more willing to consume the new products than were securely attached participants (*M* = 2.61, *SD* = 1.59), *F*(1,99) = 15.01, *p* < 0.001, supporting Hypothesis 1.

## Study 2: Risk-Taking in Various Domains

The findings from Study 1 revealed that attachment styles significantly predicted willingness to take risks by consuming new experiential products, showing that insecurely (securely) attached individuals were more (less) likely to take risks. However, doubt remains as to whether the two scenarios mimic real-life decision contexts or represent risk-taking in other domains, given that risk-taking could be domain-specific (Weber et al., 2002). Therefore, we conducted a second study to test the relationship between attachment styles and risk-taking behaviors in five, more general, domains.

### Methods

#### Participants

In this *Mturk* study, 203 adult participants (*M*_age_ = 35.02, *SD* = 10.94; 129 men; 105 are parents of children under 18^2^) were recruited online. Before the study, all participants signed an informed consent form.

#### Measures and Procedures

After signing the consent form, participants answered the 40-item domain-specific risk-taking behavior scale (Weber et al., 2002), in which eight items are used to measure each of five domains: financial, health/safety, recreational, ethical, and social decisions. For each item, participants indicated their likelihood of engaging in the provided activities on a five-point Likert scale, anchored by 1 = *very unlikely* and 5 = *very likely*. For example, the health/safety domain included the item “Not wearing a seatbelt when riding as a passenger in the front seat”; recreational risk-taking included the item “Piloting your own small plane if you could”; the ethic domain included the item “Forging a signature”; and the social risk domain included the item “Wearing provocative or unconventional clothes on occasion.” In addition, we separated financial decisions into two subdomains – gambling (4 items) and investment (4 items). Gambling included the item “Betting a day’s income at the horse races.” Investment included the item “Investing 5% of your annual income in a very speculative stock.” The average score of the 40 items formed the dependent variable (Cronbach’s α = 0.94). Higher scores indicated higher risk-taking tendencies.

As in Study 1, we measured attachment styles by the ECR scale (Brennan et al., 1998). 89 participants were classified as securely attached; 114 were classified as insecurely attached. Finally, we collected basic demographic information, such as age and gender.

### Results and Discussion

**FIGURE 1:**
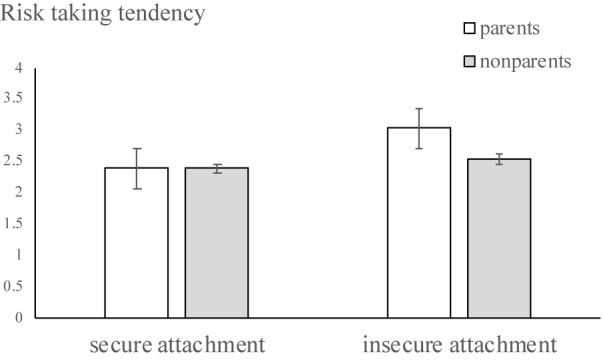
Study 2 results showing differences in risk taking tendency.

We conducted a univariate analysis to test the prediction that attachment styles (0, insecure attachment; 1, secure attachment) and parental status (0, non-parents; 1, parents) interact to predict risk-taking tendency, controlling for age and gender. As Hypothesis 2 predicted, attachment style significantly interacted with the condition of living with children, *F*(1,197) = 5.62, *p* < 0.05 (Figure 1). Simple main effect analyses showed that participants with insecure attachment were more risk-taking when there were children living in the family (*M* = 3.03, *SD* = 0.76) than when there weren’t (*M* = 2.54, *SD* = 0.72), *F*(1,197) = 20.45, *p* < 0.001. For participants with secure attachment, whether there were children living in the family, risk-taking tendencies did not differ significantly (*M* = 2.38, *SD* = 0.58 vs. *M* = 2.38, *SD* = 0.54), *F*(1,197) = 0.69, *p* > 0.05. Both main effects were significant. Supporting Hypothesis 1, insecurely (securely) attached individuals were more (less) risk-taking. Supporting the prediction of risk-sensitivity theory, when there were children living in the family, which heightened the need of parenting, individuals were more risk-taking.

When risk-taking tendency was individually tested across domains, similar interactions were found in the domains of ethics, recreation, gambling, and investment domains (marginally significant, *p* = 0.08), but not in the domains of social and health. The main effect of attachment style was significant in all the domains except for the investment domain. These results suggest that risk taking related to insecure attachment are not domain specific.

## Study 3: Risk-Taking in GM Product Consumption

Consumers associate significant risks with GM food product, which have known advantages and unknown disadvantages that might harm human health, and the natural environment. There is ample support for this claim (Scholderer et al., 1999; Grunert et al., 2001), Therefore, in a third study we used purchase intentions for GM products to measure consumers’ risk-taking behaviors. We also tested the role of safety perception as potentially mediating the relationship between attachment styles and purchase intention.

### Methods

#### Participants

This online Mturk study included 102 adult participants (*M*_age_ = 34.32, *SD* = 10.68; 64 men; 53 are parents of children under 18). Before the study, all participants signed an informed consent form.

#### Measures and Procedures

We defined safety perception as a sense of security that GM technology offers more benefits than costs (McCarty et al., 2010). Participants rated their perceptions regarding whether GM products are safe for microbes, plants, mammals, and human beings on a seven-point scale (1, *extremely unsafe*; 7, *extremely safe*). A sample item was: “Do you think it is safe to use genetic engineering techniques to modify mammals (excluding humans)?” The average score of the four items formed safety perception scores (*Cronbach’s* α = 0.91). Higher scores indicated higher safety perceptions about GM products. Safety assessment was followed by the purchase intention of GM products on a seven-point scale, anchored by 1 (*very unlikely*) and 7 (*very likely*). Finally, participants completed the 36-item dispositional attachment orientation measurement on a seven-point scale, anchored by *disagree strongly* (1), and *agree strongly* (7).

### Results and Discussion

**FIGURE 2:**
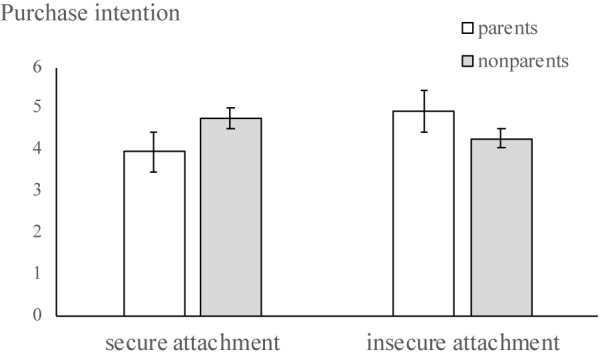
Study 3 results showing differences in purchase intention.

We conducted a univariate analysis to test the prediction that attachment style (0, insecure attachment; 1, secure attachment) interacts with parental status (0, non-parents; 1, parents) to predict purchase intention, controlling for age, and gender. The correlation between safety perception and risky purchase intention was 0.69, *p* < 0.001. As H2 predicted, attachment style significantly interacted with parental status [*F*(1,96) = 4.57, *p* < 0.05; Figure 2). Simple main effect analyses showed that for participants with insecure attachment, parents were more risk-taking (*M* = 4.94, *SD* = 1.69) than non-parents (*M* = 4.28, *SD* = 1.53), *F*(1,96) = 4.24, *p* < 0.05. For participants with secure attachment, parental status did not significantly influence their risk-taking tendencies (*M* = 3.95, *SD* = 1.62 vs. *M* = 4.76, *SD* = 1.71), *F*(1,96) = 0.93, p > 0.05. Neither main effects were significant.

### Mediation Effect of Safety Perception

**FIGURE 3:**
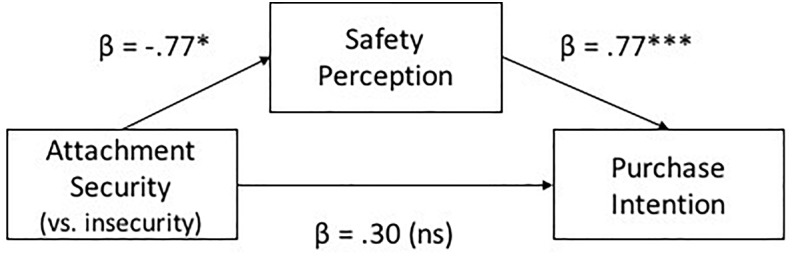
Mediation analysis of safety perception on the linkage between attachment style and purchase intention. ^∗^*p* < 0.05; ^∗∗^*p* < 0.01; ^∗∗∗^*p* < 0.001.

We hypothesized that safety perception mediates the relationship between attachment styles and GM product purchase intentions. We tested the mediation hypothesis using the bootstrapping procedure and corresponding macro (Preacher and Hayes, 2008), regressing GM product purchase intentions on safety assessment and attachment styles, with safety assessment centered as the proposed mediator. Results show that, safety perception was positively related (Figure 3; β = 0.77, *p* < 0.001) to purchase intention; when safety perception was taken into account, the direct effect of attachment security on purchase intention was insignificant (β = 0.30, *ns*). We performed 1000 bootstrap resamples. The 95% confidence interval obtained for the indirect effect of attachment styles on GM product purchase intention through safety assessment did not contain zero (0.16, 1.15). Therefore, we are confident at α = 0.05 that the corresponding increase in safety perception mediated decreased attachment security (adjusted *R*^2^ = 0.48).

The findings revealed that relative to securely attached participants, insecurely attached participants had stronger intentions to purchase GM products, and suggesting more risk seeking. Mediation analysis showed that safety perception mediated the effect of attachment styles on purchase intentions. Specifically, insecurely attached individuals perceived GM products to be safer, and were hence more likely to take risks in buying GM products. Hypothesis 3 was supported.

## General Discussion

Individuals vary in their life history strategies that are related to differences in early-life conditions (Belsky et al., 1991; Ellis et al., 2009). As an indicator of early-life conditions, the relationship between adult attachment and risk taking is straight forward in the context of life history theory, with insecurely attached individuals consistently taking more risk. Nonetheless, little is known concerning how far these risk takers can go. Echoing recent discussion that life history strategies manifest themselves into stable individual differences (Wolf et al., 2007), the current research suggests insecurely attached individuals might take more risks beyond evolutionary valid domains of risk, namely consumer behavior, and this effect is modulated by individuals’ perceived need in a foci situation. We take into account the modulation role of a life history variable, parental status, as an indicator of situational need.

Consistent with our predictions, we showed that insecurely (vs. securely) attached participants were more (vs. less) risk-taking across the domains (H1, studies 1 and 2), and this effect was moderated by parental status (H2, studies 2 and 3). Whereas being a parent led insecure-attached individuals to take more risks, it did not significantly change risk propensity of securely attached individuals. We also directly tested the psychological mechanism underlying this effect (H3), showing that the linkage between attachment security and risk-taking is mediated by individuals’ safety perception (Study 3), suggesting that relative to securely attached individuals, insecurely attached individuals are more risk-taking because they perceive a purchasing GM product to be safer. Our study explored domain specificity of the influence attachment styles and showed that in the majority of the domains (except for the investment domain), insecurely attached individuals were more risk-taking, suggesting a domain-general effect of attachment insecurity on risk taking.

Our research contributes to the literature on sensitization models (Griskevicius et al., 2011a,b, 2013), which posits that early developmental environments sensitize individuals to respond to adversity in life in different ways. The expression of fast and slow strategies is contingent on current levels of stress (Griskevicius et al., 2013). The moderation role of parental stress was conceptually consistent with past research showing that levels of stress triggered by cues of mortality, economic recession, or competitive disadvantages lead individuals to respond based on their life history strategies. For example, Griskevicius et al. (2011b) showed that people who grew up in low socio-economic status environments were more risk-taking when exposed to cues of mortality; whereas mortality cues did not increase risk taking for people from high socio-economic status environments as a child. We extend these models by suggesting that individuals with different life history strategies might perceive the same stressful situations differently. Specifically, we suggest that being a parent might be more stressful for insecurely attached individuals than those securely attached. Therefore, it triggers higher levels of stress/need in insecurely attached parents, and hence more risk-taking.

Our research found that insecurely attached individuals perceived GM products to be safer than securely attached individuals, therefore they took more risks. At first glance, this finding contradicts with commonly held beliefs that securely attached individuals should have perceived the world to be safer and explore the environment beyond one’s relationship compared with insecurely attached individuals (Bowlby, 1988; see also Green and Campbell, 2000; Waters and Cummings, 2000; Crowell et al., 2002). Our results show that this “sense of felt security” (Sroufe and Waters, 1977) is different from one’s safety perception. Echoing previous research concerning individuals’ beliefs about future predictability (Hill et al., 1997; Zhu et al., 2019), our result suggests that insecurely attached individuals, having been raised in an unpredictable environment, adopt fast life strategies that focus on short-term benefits and neglect long-term ones, and form distorted perceptions of risk/safety.

Our research has important implications for developing marketing strategies. Drawing on the effect of life history strategies on “resource scarcity” (Griskevicius et al., 2013), there has been a recent surge in consumer behavior research investigating resource scarcity from the life history perspective (for a review, see Hamilton et al., 2018). For example, Mittal and Griskevicius (2016) showed that reminding resource scarcity affected adult health care decisions, such that people who grew up poor were less interested in health coverage compared to those who grew up wealthy. This effect emerged most strongly when adults were experiencing financial threat. Corroborating these findings, we show that consumers with insecure attachment were more willing to take risks in consuming new experiential and genetically modified products. This effect emerged more strongly for parents than non-parents. It is reasonable to predict that they will react differently to limited-quantity promotions (Kristofferson et al., 2016), and to range marketing offers (Fan et al., 2018).

Our research has several limitations. First, it is correlational in nature, which could only provide preliminary evidence showing how attachment styles influence consumer risk-taking behaviors. Future research is needed to reveal the causal relationship. Drawing on social-cognitive theory, Baldwin et al. (1996) posited that most people have multiple models of relationships, but one model is more chronically accessible, depending on intensity, and frequency of corresponding relational experiences. Future research could test our finding by directly manipulating accessibility of attachment working models. Second, we tested the safety perception of GM product as a mediator, serving as a proximate cause for risk taking. Future studies could test the more ultimate reasons that account for the linkage between attachment styles and risk taking (Belsky et al., 1991). Last but not least, we generated our hypotheses drawing heavily on studies concerning perceived resource need. Nonetheless, we did not directly test perceived need, or compared need perception of securely and insecurely attached participants. Future research should directly test the implication of parenting status and adult attachment on important decisions through the micro-mediation of perceived need.

This paper provides preliminary evidence concerning how attachment style influences risk-taking among consumers, contingencies of the effect (i.e., parental status), and underlying mechanisms. Ours lends support to the significance of applying the evolutionary perspective in understanding the effect of attachment styles on customer behavior beyond the relational domain. Understanding the impact of attachment styles on consumers’ risk-taking behaviors could help marketers to segment and target potential customers, and to develop marketing strategies.

## Ethics Statement

This study was carried out in accordance with the recommendations of APA’s ethical guidelines, Research Ethical Committee of Business School, Nanjing University. The protocol was approved by the Research Ethical Committee of Business School, Nanjing University. All subjects gave informed consent prior to participation in the study. All subjects could abort the experiment at any time.

## Author Contributions

All authors listed have made a substantial, direct and intellectual contribution to the work, and approved it for publication.

## Conflict of Interest Statement

The authors declare that the research was conducted in the absence of any commercial or financial relationships that could be construed as a potential conflict of interest.

## Footnotes

1 Only secure vs. insecure dichotomies are consistent across measurement systems and are most stable across life spans (Waters et al., 2000; Crowell et al., 2002; Stein et al., 2002). Therefore, we adopted the secure vs. insecure dichotomy based on ECR scale scores, rather than using the two-dimension.

2 We asked participants to indicate their family structure by choosing on from the 6 options (single with children, single without children, married with children, married without children, life partner with children, and life partner without children). We code those who chose with children options as parents, and those who chose without children as non-parents.
